# Nutrient Intake and Nutritional Status in Adult Patients with Inherited Metabolic Diseases Treated with Low-Protein Diets: A Review on Urea Cycle Disorders and Branched Chain Organic Acidemias

**DOI:** 10.3390/nu12113331

**Published:** 2020-10-29

**Authors:** Francesco Francini-Pesenti, Giorgia Gugelmo, Livia Lenzini, Nicola Vitturi

**Affiliations:** 1 Department of Medicine-DIMED, University of Padova, Division of Clinical Nutrition, University Hospital, 35128 Padova, Italy; francescofrancini@yahoo.it (F.F.-P.); giorgia.gugelmo@aopd.veneto.it (G.G.); 2 Department of Medicine-DIMED, University of Padova, Hypertension Unit, University Hospital, 35128 Padova, Italy; livia.lenzini@unipd.it; 3 Department of Medicine-DIMED, Division of Metabolic Diseases, University Hospital, 35128 Padova, Italy

**Keywords:** urea cycle, organic acidurias, inherited metabolic diseases, low protein diet, adult patients

## Abstract

Low-protein diets (LPDs) are the main treatment for urea cycle disorders (UCDs) and organic acidemias (OAs). In most cases, LPDs start in childhood and must be continued into adulthood. The improved life expectancy of patients with UCDs and OAs raises the question of their consequences on nutritional status in adult subjects. As this topic has so far received little attention, we conducted a review of scientific studies that investigated the nutrient intake and nutritional status in adult patients with UCDs and branched chain organic acidemias (BCOAs) on LPD. Methods: The literature search was conducted in PubMed/MEDLINE, Scopus, EMBASE and Google Scholar from 1 January 2000 to 31 May 2020, focusing on nutrient intake and nutritional status in UCD and OA adult patients. Results: Despite protein restriction is recommended as the main treatment for UCDs and OAs, in these patients, protein intake ranges widely, with many patients who do not reach safety levels. When evaluated, micronutrient intake resulted below recommended values in some patients. Lean body mass resulted in most cases lower than normal range while fat body mass (FM) was often found normal or higher than the controls or reference values. Protein intake correlated inversely with FM both in adult and pediatric UCD patients. Conclusions: The clinical management of adult patients with UCDs and BCOAs should include an accurate assessment of the nutritional status and body composition. However, as little data is still available on this topic, further studies are needed to better clarify the effects of LPDs on nutritional status in adult UCD and BCOA patients.

## 1. Introduction

Inherited metabolic diseases (IMDs) are errors of metabolism due to enzymatic defects in carbohydrates, protein, and lipid metabolism. They are very different among each other, with a wide spectrum of clinical presentation and severity, with life expectancy varying from days to general population’s expectancy.

Urea Cycle disorders (UCDs) are inherited diseases due to a loss of function of one of the enzymes related to urea cycle: deficiency of carbamoyl phosphate synthetase 1 (CPS1D, MIM#237300), ornithine transcarbamylase (OTCD, MIM#311250), argininosuccinatesynthetase (ASSD, MIM#215700), argininosuccinatelyase (ASLD, MIM#207900) and arginase (ARG1D, MIM#207800), the mitochondrial ornithine-citrulline antiporter (ORC1D, MIM#238970) and the CPS1-activating enzyme N-acetylglutamate synthase (NAGSD, MIM#237310) [1]. The overall incidence of UCDs is about 1:35,000 births, and they are all autosomal recessive with the exception of OTC deficiency that is X-linked. The loss of function of the urea cycle causes the inability to excrete ammonium produced during protein catabolism. The resulting hyperammonemia is toxic to the brain.

Branched-chain organic acidemiasi (BCOAs) (or “classic” acidemias) are inherited diseases caused by a loss of function of enzymes related to the catabolism of branched chain amino acids (BCAAs) [2]. The more frequent BCOAs are isovaleric aciduria (IVA, MIM#243500), propionic aciduria (PA, MIM# 606054) and methyl malonic aciduria (MMA, MIM#251000). IVA is caused by a deficiency of isovaleryl-CoA dehydrogenase (EC 1.3.99.10), PA by a deficiency of the propionyl-CoA carboxylase (PCC EC 6.4.1.3), and MMA by a deficiency of methylmalonyl-CoA mutase (MUTEC 5.4.99.2). All BCOAs cause an accumulation of toxic organic acids. They are multi-systemic diseases, with neurological damages associated with organ involvement linked to the single form (i.e., cardiac involvement in PA, renal involvement in MMA). These diseases often have a more severe neonatal form and a chronic progressive, late onset form [2].

In UCDs, long term suggested therapy consists in drugs increasing waste nitrogen excretion, supplementation of arginine and/or citrulline and diet [1]. For MMA and PA, L-carnitine, vitamin B12 (in cobalamin-responsive MMA) and drugs increasing waste nitrogen are suggested in long term management; for IVA, glycine and carnitine supplementation [3]. Presently, thanks to neonatal screening, many patients reach adulthood and so their therapy is maintained for life. For this reason, the focus of IMDs clinicians is changing from acute, life threatening events (i.e., stroke-like episode) to chronic complications, caused by both the disease itself and therapy’s adverse events. While there is a wide corpus of data describing drug’s adverse events, long-term effect of dietary treatment is largely unknown.

Low protein diet (LPD) is one of mainstays of treatment [1] in UCDs. In the acute phase is essential to avoid protein intake, and parenteral nutrition is needed to prevent catabolism [1]. Glucose and lipids must be given for 24–48 h, with no proteins or amino acids (AA), or with essential amino acids (EAAs) as supplementation [3]. The recommendations for the long-term management of patients with UCDs are based on reducing the intake of protein in general and supplementing with citrulline or arginine (except in arginase deficiency to feed the cycle), detoxifying with nitrogen reducing agents depending on the metabolic balance (and which UCDs), and activating urea cycle with N-Carbamylglutamate (when possible). It may be necessary to ensure that the patient receives optimal amounts of EAAs and micronutrients by substitution [1,4]. Similarly, in BCOAs, LPD is proposed to minimize the production of toxic metabolites of organic compounds [4].

In IVA, disease caused by a defect in the degradation of the essential amino acid leucine, the treatment is a balance between a deficiency of leucine in the nutrition and the accumulation of toxic intermediates when the intake of leucine is too high (varying between catabolism and anabolism). Thus, it may be necessary to supplement with synthetic food enriched in amino acids without leucine.

In propionic and methylmalonic acidemia, the balance is even more difficult to optimize, since the defects in these disorders are in the degradation of the four EAAs valine, isoleucine, methionine and threonine (plus fatty acids, which are degraded into propionylCoA). It may be necessary to substitute with synthetic food without the four amino acids. There is a general risk of a deficiency of these four amino acids when the protein reduction is strict to avoid accumulation of toxic metabolites. As an example, a low concentration of methionine will lower the concentration of S-adenosylmethonine, the essential methyl group donor in the metabolism, compromising several synthetic pathways in the body. During severe catabolic states, there is also a risk of ammonia intoxication that has to be treated with ammonia scavenger drugs [4].

However, an inadequate protein intake can compromise growth and adversely affect several body structures and functions [5]. Because of this, some authors [1] recommended that the dietary protein content should be based on the safe level established by Food and Agriculture Organization/World Health Organization/United Nations University (FAO/WHO/UNU) [5], providing high biological value proteins to ensure the adequate supply of all EAAs. Since the protein intake in UCDs must be carefully individualized, and several patients show a poor tolerance to protein foods, the safe level cannot be often achieved and EAA supplementation have to be employed [6]. Moreover, the therapy with high doses of nitrogen scavenger sodium phenylbutyrate (PB) can reduce BCAA plasma level, and a supplementation can be required [7]. When oral nutrition does not allow to meet nutritional needs, the enteral feeding is recommended [2].

Patients on LPD are at risk from deficiency of several micronutrients, including vitamin B12, iron, calcium, zinc and copper [8]. Given the improved life expectancy of patients with UCDs and BCOAs, we performed a review of the literature to assess the adequacy of nutrient intake and nutritional status in adult patients with UCDs and BCOAs on LPD.

## 2. Materials and Methods

We conducted a review of the scientific literature by customized search strategy in PubMed/MEDLINE, Scopus, EMBASE and Google Scholar (papers published between 1 January 2000 and 31 May 2020) to identify studies that investigated nutritional aspects in patients with UCDs or BCOAs: we focused on nutrient intake (i.e., vitamin, micronutrients) and nutritional status (i.e., body composition, energy expenditure) in adult patients.

Animal and biochemical studies were excluded, as well as articles published in languages other than English.

## 3. Results

We recorded 140 papers published from 1 January 2000 to 31 May 2020. We excluded 128 references that did not match with the criteria of this review, and then, 12 papers were considered. The main studies investigating nutrient intake, energy expenditure and body composition in UCD and BCOA adult patients are summarized in Table 1.

### 3.1. Nutrient Intake

In 2012 Adam et al. [9] described dietary practices in British patients with UCDs, comparing food intake with FAO/WHO/UNU recommendations. Seventeen dietitians from British hospitals were sent a questionnaire about the dietary management of UCDs. Questionnaires were returned from 175 patients treated with LPD, of which 52 were aged > 16 years (defined “adult patients”). In adult patients, total protein intake ranged from 0.4 to 1.2 g/kg body weight/day (median 0.8), and FAO/WHO/UNU safe level varied from 0.84 to 0.87 g/kg/day. Eight adult patients (6.5%) were prescribed EAA supplementation. In a later study [8], the same authors involved 41 European IMD centers in a larger survey. Questionnaires were returned from 9 countries, representing 41 centers across Europe and providing data on 464 patients with UCDs, of which 137 aged > 16 years. Authors did not report data of protein intake separately for adults but, overall, they concluded that UCD nutritional practices (both in natural protein intake and in EAA supplementations) across European countries varied in a wide range.

Hook et al. [10] examined the protein and calorie intakes of 45 adult and 49 pediatric UCD patients who participated in clinical trials of glycerol phenyl butyrate. Authors pooled and analyzed data from 4 clinical studies in which only UCD glycerol phenyl-butyrate eligible patients were included. In adult population, actual dietary data was assessed by dietary diaries and by physicians’ prescription; then, authors compared these data to published UCD dietary guidelines and nutritional surveys. Mean protein intake in adult patients resulted higher than UCD recommendations but lower than normal population values (U.S. Recommended Daily Allowance (RDA) [17] and National Health and Nutrition Examination Survey (NHANES) [18,19]). Energy intake (both prescribed and actual) was reported lower than UCD treatment guidelines and also lower than RDA and NHANES references. Authors concluded that protein and energy intakes both in adult and pediatric UCD patients differed from UCD dietary recommendations, suggesting that future guidelines may consider nutritional practices observed in these patients.

Martin-Hernandez et al. [11] investigated protein intake from detailed diet histories, anthropometrics data such as weight, height, body mass index (BMI); laboratory tests (hemoglobin, folic acid, vitamin B12, and plasma amino acid profile) in 15 OA adult patients (median age of 23.5 years, range 18–48). The mean amount of protein was 0.72 g/kg per day (0.5–1.09), and no patients took synthetic amino acid supplements; 2 PA patients required overnight enteral feeding, 4 patients received energy supplements during the day. Anthropometrics resulted in normal BMI in 9 patients, high in 3, and low in 3 (the three underweight with normal values of Hb, folic acid, vitamin B12 and essential amino acids), height below the 3rd centile in 4/7 patients with MMA and 1 with PA. Laboratory tests showed all patients had normal values of serum folic acid; serum B12 was in the normal range in 13 patients, and EAAs were below the normal range in 6 patients.

Manoli et al. [12] studied 61 MMA patients (age range: 2.5–35 years) observing that they tolerated close to the recommended daily allowance (RDA) from natural protein. Nevertheless, 65% of patientswas consuming various amounts of special MMA formulas in addition to natural protein. In the subgroup of adult subjects with methyl-malonyl-CoA mutase deficiency (*n* = 6), total protein intake was 0.81 ± 0.28 g/kg/day, of which 0.76 ± 0.21 g/kg/day was natural protein (94.6 ±27.8% of RDA) [17].

Most recent data regarding MMA nutritional management were reported by Pinto et al. [13], with a survey across 53 European IMD centers which were following in total 80 patients with MMA vitamin B12 responsive (MMAB12r) and 215 patients with MMA vitamin B12 non-responsive (MMAB12nr). Data from centers were analyzed and divided into 5 different age ranges (0–6 months 7–12 months; 1–10 years; 11–16 years; >16 years). For MMAB12r patients older than 16 years prescribed natural protein was below the (WHO/FAO/UNU 2007) safe levels of protein intake in centers using Precursor Free Amino Acids (PFAAs) (0.6 g/kg/day) and higher than safe levels in centers not using PFAAs (1g/kg/day). In MMAB12nr patients older than16 years, prescribed natural protein intake was lower than (WHO/FAO/UNU 2007) safe levels of protein intake both in centers using PFAAs (0.6 g/kg/day) and in centers not using PFAAs (0.8 g/kg/day) [20]. PFAAs were prescribed by 77% of centers managing 81% of patients. In MMAB12nr patients, 42% required tube feeding.

### 3.2. Energy Expenditure and Body Composition

The first studies on energy expenditure in patients with OAs were carried out on pediatric patients, showing conflicting data. Feillet et al. [21] in 14 subjects with alterations of propionate metabolism observed that resting energy expenditure (REE) measured by indirect calorimetry (IC) was 80 ± 18% of that predicted by Schofield equation. Otherwise, van Hagen et al. [20] in 5 children with PA reported that the measured REE was 108 ± 11% compared with the value predicted by the Schofield equation.

Hauser et al. [22] measured REE using IC and body composition by dual-energy X-ray absorptiometry (DXA) in 29 patients with isolated MMA (children = *n*. 22; adults = *n*. 7). In patients aged >18 years, BMI Z-score was −1.5, fat mass (FM) was 23%, and measured REE was 78% ± 11% of value predicted by the Harris–Benedict equation (*p* = 0.004).

Evans et al. [14] examined relationships between dietary intake and body composition in 75 children and adults with UCDs and OAs. Data were not analyzed separately by age. Median percentage fat mass (%FM), measured using bioimpedance, was 17.6% in IVA, 20.7% in MMA/PA, 19.4% in UCDs. A significant negative correlation was observed between %FM and total protein intake in IVA, MMA/PA, and UCDs. Similarly, among UCD patients on LPD from natural sources only, higher protein intake was correlated with lower %FM.

Brambilla et al. [15] evaluated lean body mass (LM) and fat body mass (FM) by DXA analysis in 8 patients with ASL (adult *n* = 5) and in 9 patients with other UCDs (adult *n* = 5). Total body and trunk fat mass (FM) z-scores were ≤+1 SD for age and gender related cut-off in all patients [23]. Body lean mass (LM) was <−2 SD in 5 subjects (1 ASL deficiency and 4 “other UCDs”), while average leg LM was <−2 SD in 8 subjects (4 ASL deficiency and 4 “other UCDs”). No significant differences in body composition parameters were reported between the groups and between children and adults.

In a preliminary study [16], we studied 17 adult IMD patients (age range: 19–34 years), 9 with UCDs and 8 with OAs. Nutrient intake was assessed using two non-consecutive 24 h recalls, and body composition by bioelectric impedance and plasma AA profile was evaluated.

Mean calorie and protein intakes were found to be, respectively, 24.1 ± 6.4 kcal/kg/day and 0.67 ± 0.23 g/kg/day, both below FAO/WHO/UNU recommendations [5]. The food intake evaluation revealed an intake below recommended values of at least one of the following nutrients: calcium, magnesium, potassium, zinc, copper, manganese, iodine, and vitamin B12. In all patients, plasma EAA levels were within normal limits, and bioelectrical parameters (resistance and reactance) were in the normal range for Italian adult population [24]. Mean BMI was 24.2 ± 4.0 kg/m^2^ (range: 18–32). Seven patients (40%) were overweight (BMI ≥ 25 kg/m^2^).

## 4. Discussion

In recent years, advances in medicine and the availability of new effective therapies have improved the survival of patients with IMDs, and the transitional care to adulthood has become a new challenge for physicians and public health systems [25]. Sometimes, the treatment includes dietary measures that must be maintained over time. This is the case of UCDs and BCOAs that require restriction of protein intake throughout life, raising concerns about growth in pediatric patients and about nutritional status and maintenance of lean body mass in adults. It is known that a prolonged protein intake below recommended values leads to EAAs deficiency, compromising the normal protein synthesis in several tissues and reducing the intake of micronutrients contained in protein-rich foods [16].

In early childhood, dietary protein intake is associated with growth trajectories [26], and a shortage of micronutrients can, in turn, negatively affect growth [27]. In adults, LPD are widely employed in the treatment of patients with chronic kidney diseases, leading to a reduction of LM [8] and increasing the risk of death [28]. Despite its importance, the study of nutritional consequences of LPD in UCD and BCOA subjects has so far received little attention. For this reason, it is difficult to draw an exhaustive picture of the effects of LPD on the nutritional status, especially for the adult patients, and sometimes it is useful to refer to studies on pediatric patients. Data obtained from surveys evidenced a wide range of protein intakes in UCD and BCOA subjects, both in children and in adults, with many patients who do not reach safety levels and are therefore at risk of malnutrition [9,13].

To prevent the protein deficiency, EAA supplements are sometimes used, but the dosage administered differs among the centers, and guidelines are not yet clear about this. Often EAA supplements do not contain enough micronutrients, so these should be provided separately to prevent their deficiency. Micronutrient plasma levels were investigated in very few studies, reporting conflicting data. When evaluated in BCOA pediatric patients, dietary intakes of sodium, potassium, and magnesium were lower than recommended levels while micronutrient intake was in the range, except for zinc that was lower in subjects > 10 years of age [29]. However, in our preliminary study, we found that the calcium, magnesium, potassium, zinc, copper, manganese, iodine, and vitamin B12 intakes were on average below recommended values [16], suggesting that micronutrient status should be regularly investigated in all IMD patients treated with LPD. In children with BCOAs, mean iron and zinc plasma levels were reported below normal range [29]. In BCOA adults, folates were reported normal, and vitamin B12 was below the normal range only in few cases [11]. However, it is difficult to compare data from these studies because some authors evaluated the dietary intake of micronutrients and other authors their plasma level. Moreover, micronutrient plasma level is not always a good biomarker of adequacy [30], and the estimation of habitual dietary intake in a study population is often inaccurate [31]. Specifically designed studies using appropriate biochemical indicators are needed for assessing the true prevalence of vitamin and mineral deficiencies in UCD and BCOA patients.

Despite few studies analyzed body composition in UCD and BCOA patients, it is interesting to note that, while the mean LM resulted lower than normal range, FM was often found normal or higher than the controls or reference values both in pediatric and in adult UCD and BCOA subjects [12,14,15]. Moreover, it was reported that protein intake correlated inversely with FM both in UCD adult and pediatric patients [14]. In accordance with FM data, BMI in UCD and BCOA subjects is in the normal range in most cases, and overweight is not an uncommon condition [11,15,16]. However, these studies used different methods to assess body composition (BIA or DEXA) and lacked a control group of healthy subjects.

When calorie intake in UCD and BCOA adult subjects was evaluated, it was found lower than recommended [10,16]. REE measured using IC resulted reduced only in ASL deficiency patients but not in other UCD subjects. Although no study has so far assessed the level of physical activity of these patients, its reduction can be postulated as the main cause of increased BMI and FM. In many adult UCD and BCOA patients, the FM seems well represented, and the BMI is sometimes beyond normal values, suggesting that an increase of physical activity is often desirable as well as the monitoring of cardiovascular risk parameters also considering the improved life expectancy in IMDs.

The observation that protein intake was inversely correlated with lower %FM [14] rises some interesting considerations. Several factors can explain why a low protein diet can promote fat mass accumulation. First, a higher protein intake increases thermogenesis and satiety compared to diets of lower protein content [25]. Second, adequate consumption of dietary protein is critical for the maintenance of muscle mass [25]. Recently, the “protein leverage” was proposed as model of food intake regulation [25]. This model posits that the reduction of protein intake causes the overconsumption of fats and carbohydrates (hence total energy) with a low proportion of energy from protein and vice-versa. In addition, we speculate that the increase of carbohydrate consumption during metabolic stress periods (e.g., infections) could promote the accumulation of fat mass, even if evidence on this regard is still lacking.

In conclusion, protein restriction is recommended as the main treatment for UCDs and BCOAs, but protein intake ranges widely, both due to the different individual tolerance and to prescriptive differences between centers. The clinical management of these diseases in adult patients should include a periodic and accurate assessment of nutritional status, including routinely parameters that can investigate body composition, metabolic risk, and micronutrient deficiency (i.e., BIA, plasma levels of micronutrients, and EAAs). Since there are still few data on the nutritional status of adult patients on a low protein-based diet, further studies on this topic are needed.

## Figures and Tables

**Table 1:** Summary of studies investigating the nutrient intake and nutritional status in patients with branched chain organic acidurias and urea cycle disorders treated with low protein diets.

Study	Disease	Number of Patients, Age and Sex	Study Design	Nutritional Aspects Investigated	Results
Adam et al. (2013) [6]	NAGS deficiency CPS1 deficiency OTC deficiency Citrullinemia ASA Arginase deficiency	464 patients > 16 year: *n* = 137 (30%)	Cross-sectional data from 41 European IMD centers collected by questionnaire	Patients’ dietary treatment: prescribed natural protein; EAA and BCAA intakes; use of enteral tube feeds; oral energy, vitamin, mineral and EAA supplements and composition of emergency regimens. Data on nutritional and biochemical monitoring.	The prescribed median total protein intake per kg body weight decreased with age across all disorders. Total protein intake was quite variable between countries. EAAs were prescribed for 38% [*n* = 174] of the patients. 3% patients received additional BCAA supplements.
Adam S et al. (2012) [9]	NAGS deficiency CPS deficiency OTC deficiency Citrullinemia ASA Arginase deficiency	175 patients >16 year *n* = 52 (30%) of which 31 with OTC deficiency.	Cross-sectional, questionnaires from seventeen dietitians from major UK hospitals	Patients’ dietary treatment: prescribed natural protein; EAA and BCAA intakes; use of enteral tube feeds; oral energy, vitamin, mineral and EAA supplements and composition of emergency regimens. Data on nutritional and biochemical monitoring.	Adult protein prescription ranged 0.4–1.2 g kg^−1^ day^−1^ (40–60 g day^−1^). 30% were given EAAs, prescribed for: low plasma quantitative EAAs (*n* = 13 centers); inadequate natural protein intake (*n* = 11) and poor metabolic control (*n* = 9). 3% were given BCAA supplements. Oral energy supplements were prescribed in 17% of cases.
Hook D et al. (2016) [10]	UCDs	45 adult (mean age 33 ± 13 years, range 18–75) and 49 pediatric UCD patients	Observational study	Data from 4 clinical studies were pooled and analyzed (only patients eligible to phenyl-butyrate treatment). For adults, dietary data detected by 3-day diet histories (collected weekly for a total of 4 weeks) and by their physicians’ treatment prescription. Dietary data were compared to UCD recommendations and to normal population values.	In adults, mean protein intake was comparable to their medical prescription; it was higher than UCD recommendations but lower than RDA and NHANES values. Calorie intake (both prescribed and actual) was lower than UCD recommendations, RDA and NHANES
Martín-Hernández et al. (2009) [11]	MMA B12- unresponsive MMA B12-responsive IVA PA	15 adult patients (median age 23.5 years, range 18–48)	Retrospective study	Protein intake from detailed diet histories; anthropometrics: weight, height, body mass index; laboratory tests (hemoglobin, folic acid, vitamin B12, and plasma AA profile; BMD assessed by z-score.	Mean amount of protein: 0.72 g/kg per day (0.5–1.09). No patients took synthetic AA supplements. *N* = 4 patients received energy supplements during the day. *N* = 2 PA patient required overnight enteral feeding. Normal BMI in *N* = 9, high in *N* = 3, low in *N* = 3; the 3 patients underweight had normal values of Hb, folic acid, vitamin B12 and EAAs. Height below the 3rd centile in 4/7 patients with MMA and 1 with PA. All had normal values of Hb and folic acid. EAAs were below the normal range in 6 patients. BMD: demineralization in 6/8 patients (3 PA and 3 MMA), 2 osteoporosis and 4 osteopenia. One female patient with PA presented severe osteoporosis.
Manoli et al. (2016) [12]	Isolated MMA	61 patients (mean age 13.3 ± 9.1 years; MMA mut *n* = 46; MMA cblA *n* = 9; MMA cblB *n* = 6; MMA mut adult patients *n* = 6; total adult patients’ number was not specified)	Cross-sectional	Anthropometrics, body composition measurements (FM, FFM) and BMD using dual energy X-ray absorptiometry were correlated with diet content (a 3-day food record and a detailed dietary history obtained by a research dietitian, using Nutrition Data System for Research) and disease-related biomarkers (routine laboratory investigations, metabolites).	Patients with MMA tolerated close to the Recommended Daily Allowances (RDA )of complete protein (mut0: 99.45 ± 32.05% RDA). 85% received medical foods, the protein-equivalent in which often exceeded complete protein intake (35%). Medical food consumption resulted in low plasma valine and isoleucine concentrations, prompting paradoxical supplementation with these propiogenic AAs. Weight and height–for age Z-scores correlated negatively with the leucine/valine intake ratio.
Pinto et al. (2019) [13]	MMA vitamin B12 responsive MMA vitamin B12 non-responsive	Questionnaires were returned from 53 centers. MMA vitamin B12 responsive *n* = 80 patient MMA vitamin B12 non-responsive *n* = 215 patients	Cross-sectional survey	A questionnaire sent to European IMD centers about nutritional management of MMA. Data were analyzed by different age ranges (0–6 months; 7–12 months; 1–10 years; 11–16 years; >16 years).	MMAB12r patients >16 years: - Centers using PFAAs prescribed natural protein (0.6 g/kg/day) lower than WHO/FAO/UNU 2007 safe; - centers not using PFAAs prescribed natural protein (1 g/kg/day) higher than safe levels MMAB12nr patients > 16 years: both centers using PFAAs (0.6 g/kg/day) and centers not using PFAAs (0.8 g/kg/day) prescribed natural protein intake lower than WHO/FAO/UNU 2007 safe levels. PFAAs were prescribed by 77% of centers managing 81% of patients. In MMAB12nr patients 42% required tube feeding.
Evans M et al. (2017) [14]	IVA MMA PA UCDs MSUD	Retrospective longitudinal *n* = 75 patients Prospective longitudinal *n* = 21 patients	Retrospective longitudinal data of growth and dietary intake Prospective longitudinal data of growth, dietary intake, and body composition	Longitudinal data on dietary intake and growth of patients born between 1976 and December 2014 from medical and dietetic clinic records (dietary recall, food diaries, and dietary history).	Total natural protein intake decreased with age in all patients yet met or exceeded the FAO/WHO/UNU 2007 safe level except for UCD patients at 14 years, whose median protein intake was 0.8 g/kg/day FAO/WHO/UNU 2007 recommendation of 0.9 g/kg/day). UCD patients: 12/44 reported 1 or more episodes where protein intake was less than recommended. The median energy intake over the data collection period was 141–192%. At baseline measurement, the median FM was 19.4%. Dietary variables were not consistently associated with height or weight z-score. Significant negative correlation between total protein intake and BMI.
Brambilla et al. (2019) [15]	ASA deficiency OTC-deficiency ASS-deficiency	ASA *n* = 13 patients OTC *n* = 20 patient 15 children and 18 adult patients	Observational	Anthropometric parameters, body composition, risk of MS, and REE, both by IC and predictive equations.	Total body and trunk FM z-scores were ≤+1 SD for age and gender related cut-off in all patients. Body LM was <−2 SD in five subjects (1 ASA and 4 “other UCDs”). Average leg LM was <−2 SD in eight subjects (4 ASA and 4 “other UCDs”).
Gugelmo et al. (2020) [16]	UCDs OAs	Adult UCD patients *n* = 9 Adult OA patients *n* = 8 Median age 26 ± 7 years	Observational	Anthropometric parameters, body composition (BIA analysis), nutrient intake (24-h recalls for 2 non-consecutive days) compared to EFSA DRVs. Laboratory plasmatic levels (albumin, AAs profile, ammonia, transaminases, glucose, TG, TC, HDL-C and LDL-C).	Patients had been on LPDs for 25.4 ± 6.0 years. Median BMI = 24.2 ± 4.0 kg/m^2^ (18-32); 7/17 patients (40%) had a BMI > 25 kg/m^2^. Median FFM = 73 ± 8.5% (58.8–85.3); phase angle 6 ± 0.9 (4.6–7.5) Median energy intake = 24.1 ± 6.4 kcal/kg/d (16.2–37.5), about 9 kcal/kg/d lower than DRVs. Median protein intake = 0.63 ± 0.19 g/kg/d (0.47–1.24), below WHO/FAO/UNU 2007 safe levels. Intake of minerals and micronutrients was reported below recommended values for calcium, magnesium, potassium, zinc, copper, manganese, iodine, and vitamin B12. Plasmatic EAA levels were in the normal range in all patients. Lipid plasma profile evidenced TG 1.1 ± 0.5 mmol/L (0.4–2.0), C-HDL 1.2 ± 0.3 mmol/L (0.7–1.9), C-LDL 2.6 ± 0.7 mmol/L (1.4–3.8).

Abbreviations: NAGS, N-acetyl glutamate synthase; CPS1, carbamoyl phosphate synthetase 1; OTC, ornithine transcarbamoylase; ASA, arginine succinic aciduria; MMA, methylmalonic acidaemia; IVA, isovaleric aciduria; PA, propionic acidaemia; UCDs, urea cycle disorders; MSUD, maple syrup urine disease; AS, argininesuccinate synthetase; MMAB12r, Methylmalonic Acidaemia B12 responsive; MMAB12nr, Methylmalonic Acidaemia B12 non responsive, REE, resting energy expenditure; MS, metabolic syndrome; AAs, Amino Acids; BCAAs, Branched Chain Amino Acids; PFAAs, Precursor Free Amino Acids; EAAs, Essential Amino Acids; FM, Fat Mass; FFM, Free Fat Mass; LM, Lean Mass; EFSA DRVs, European Food Safety Authority Dietary Reference Values; LPDs, Low Protein Diets; BMI, Body Mass Index; TG, Triglycerides; TC, Total Cholesterol; C- HDL, HDL Cholesterol; C-LDL, LDL Cholesterol; BMD, Bone Mineral Density; CblA, Cobalamin A; CblB, Cobalamin B, IMD, Inherited Metabolic Disorders; BIA, Bioelectrical Impedance Analysis; IC, Indirect Calorimetry, SD, Standard Deviation.
